# Miracle Cure: The Creation of Antibiotics and the Birth of Modern Medicine

**DOI:** 10.3201/eid2501.181184

**Published:** 2019-01

**Authors:** Keith W. Hamilton

**Affiliations:** The University of Pennsylvania, Philadelphia, Pennsylvania, USA

**Keywords:** Antibiotics, antibiotic development, history of antibiotics, antimicrobial resistance, antimicrobial drugs

Miracle Cure: The Creation of Antibiotics and the Birth of Modern Medicine (Figure) engagingly describes what is arguably the most significant development in the history of medicine: antibiotics. The book chronicles captivating accounts from the conception of the germ theory of disease and the scientific discovery of these life-saving medications to the interplay among the countless contributing idiosyncratic and imperfect individuals and organizations involved, including the pharmaceutical titans. With the contemporary emergence and spread of antibiotic resistance, the book’s message is timely and poignant.

**Figure:**
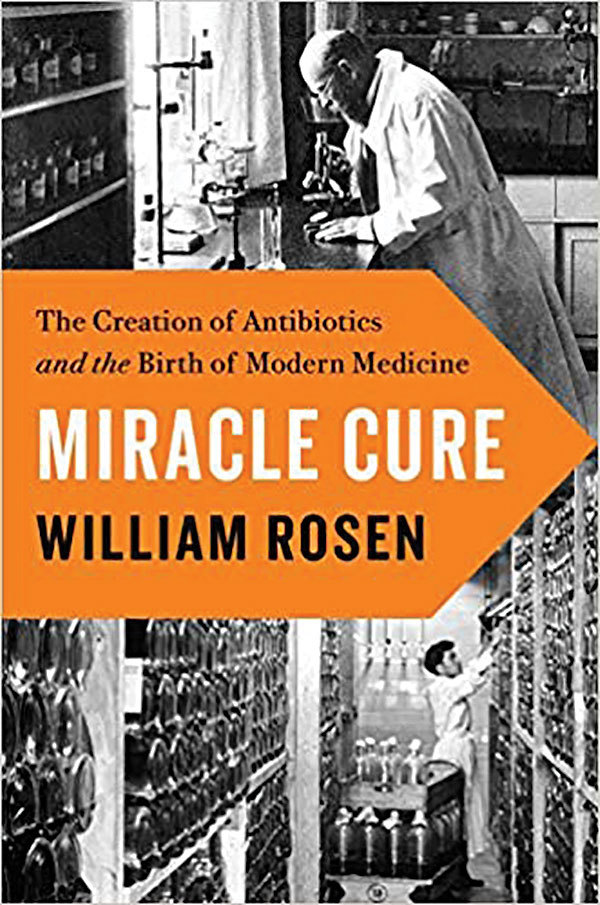
Miracle Cure: The Creation of Antibiotics and the Birth of Modern Medicine

*Miracle Cure* is relevant for both the science enthusiast and the science novice. Although slightly tangential at times in its background narratives, it intertwines the creation of antibiotics as intricately tied to the evolution of modern medicine and paints both as not just a product of science but as a culmination of economic, political, and social influences. In so doing, it sheds a human light on the discovery and production of antibiotics.

Many medical professionals and nonmedical persons envision the discovery of antibiotics, especially penicillin, as a providential occurrence that revolutionized previously unenlightened medical practices characterized by bloodletting and poisoning. However, *Miracle Cure* paints them more accurately as products of the iterative process of scientific discovery in an attempt to improve public health and, in some instances, generate personal profit and fame. The lifesaving properties of antibiotics are a secondary theme to the real-life problems overcome, the challenging ethics decisions made, and the delicate scientific egos bruised along the way.

For readers seeking a book that provides insight into the current public health crisis of antibiotic resistance, *Miracle Cure* does not provide answers or potential solutions, nor was it intended to do so. However, it goes beyond the most apparent impacts antibiotics have had on human health to explore the less publicized effects of antibiotics on regulatory agencies, drug marketing, physician–pharmaceutical industry relationships, research study design, and the practice of medicine.

One of the book’s most important arguments is that antibiotics have forced us to calibrate and recalibrate our idea of medication safety as we transitioned from unregulated mixtures of strychnine, mercury, and arsenic for which the adverse effects were inextricably tied to the morbidity and mortality of underlying disease to tightly regulated, carefully calibrated antibiotics for which safety and efficacy were the norm. *Miracle Cure* demonstrates that this shift, facilitated by a mix of altruism and greed, caused the “[p]rescription of antibiotic without a specific cause” to reach “disturbing proportions.” The book is a fascinating and important read that translates to a deep understanding of the history of antibiotic development leading up to the current state and its problems.

